# Multilayer all-dielectric metasurfaces expanding color gamut

**DOI:** 10.1515/nanoph-2024-0258

**Published:** 2024-06-26

**Authors:** Xin Gu, Jiaqi Li, Zhouxin Liang, Bo Wang, Zhaoxiang Zhu, Yujie Chen

**Affiliations:** State Key Laboratory of Optoelectronic Materials and Technologies, School of Electronics and Information Technology, 26469 Sun Yat-Sen University, Guangzhou, China; State Key Laboratory of Optoelectronic Materials and Technologies, Guangdong Provincial Key Laboratory of Optoelectronic Information Processing Chips and Systems, School of Electronics and Information Technology, 26469 Sun Yat-Sen University, Guangzhou, China

**Keywords:** metasurfaces, structural color, silicon-rich silicon nitride, multipole expansion, high-order resonances

## Abstract

Structural color, arising from the interaction between nanostructures and light, has experienced rapid development in recent years. However, high-order Mie resonances in dielectric materials often induce unnecessary sub-peaks, particularly at shorter wavelengths, reducing the vibrancy of colors. To address this, we have developed a multilayer dielectric metasurface based on silicon-rich silicon nitride (SRN), achieving expanded color gamut through precise refractive index matching and suppression of high-order resonances. This strategy introduces more design dimensions and can reduce the complexity of material deposition. It enables the generation of vibrant colors in a 3 × 3 array, with a resolution of approximately 25,400 dpi, demonstrating its potential applications in displays.

## 1 Introduction

Color plays a vital role in the preservation and transmission information [1], [2], [3]. Dyes and pigments are commonly used for coloration by absorbing and reflecting light [4]. However, they often result in dull colors, low resolution, and limited color gamut. Additionally, these substances exhibit chemical instability and are susceptible to fading when subjected to high temperatures or intense ultraviolet (UV) radiation [5]. To overcome these limitations, plasmonic and dielectric metasurfaces have been proposed for color generation [6], [7], [8]. The interaction of light with plasmonic nanostructures [9], [10], [11], such as gratings [12], [13], nanogaps [14], [15], [16], and nanoparticles [17], [18], [19], can produce vibrant colors covering the entire visible spectrum and enable high-resolution color printing beyond the diffraction limit, achieving resolutions of up to 100,000 dpi [20], [21], [22]. However, colors generated by plasmonic nanostructures are limited by their high optical losses, which hinder the production of efficient and highly saturated colors [23], [24], [25], [26], [27], [28]. In contrast, all-dielectric metasurfaces with high refractive index and low optical losses [29], [30], [31], [32], [33], [34], which exhibit more vivid colors and can significantly expand the color gamut, have gained increasing attention [34], [35], [36], [37], [38], [39], [40].

The key to achieving high-performance structural colors is to controlling the Mie resonances of each dielectric unit [41], [42], [43], [44], [45], [46]. Combining a fundamental magnetic dipole resonance with a Mie lattice resonance is a feasible approach for achieving high-purity colors [47]. By embedding an index-matched silicon nitride (Si_3_N_4_) layer between the antireflective coated substrate and amorphous silicon nanopillars, a sharper optical resonance can be observed corresponding to the realization of Kerker’s conditions [48]. Moreover, multiple resonances consisting of silicon dioxide (SiO_2_), titanium dioxide (TiO_2_), and Si_3_N_4_ can realize full modulation of Mie resonance modes [49]. To evaluate the universalities of various methods for modifying resonances, Table 1 summarized related comparisons with five key parameters – the spatial resolution, the reflection, the fabrication difficulty, the gamut area in the international commission on illumination (CIE) color diagram and the full width at half maximum (FWHM). This thorough analysis facilitates a comprehensive evaluation and comparison of the performance of diverse dielectric materials with respect to the discussed parameters. The geometry and refractive index distribution of nanopixels still provide ample design dimensions for improving performance.

**Table 1:** Summary of representative metasurface properties and performance metrics.

Constituent material	Reflection	Resolution	FWHM	Color gamut	Manufacturability	Ref.
Si_3_N_4_	∼75 %	10,160 dpi	∼20 nm @ 660 nm	∼sRBG	★	[47]
Si_3_N_4_\α-Si	∼40 %	100,000 dpi	∼80 nm @ 546 nm	120 % sRBG	★★	[48]
Si_3_N_4_\TiO_2_\SiO_2_	88.1 %	18,000 dpi	34 nm @ 490 nm	128 % sRBG	★★★	[49]
SiN_*x*_\SRN\SiO_2_	77.7 %	25,400 dpi	25 nm @ 468 nm	122 % sRBG	★★	This work

Si_3_N_4_ is attained with low pressure plasma chemical vapour deposition (LPCVD), SiN_*x*_ is attained with inductively coupled plasma chemical vapour deposition (ICP-CVD). The ★ in this rating system represents the level of complexity of a process, with ★ indicating simplicity and ★★★ indicating high complexity.

To suppress the excitation of high-order Mie resonances and improve efficiency, we propose a multilayer all-dielectric metasurface composed of SiO_2_, SRN, and silicon nitride (SiN_*x*_) layers. The design of proposed multilayer SRN-based metasurface should satisfy the critical requirements: (i) high refractive index with large tuning range for SRN material [49], [50], [51], [52]; (ii) the embedding refractive index-matching layers for air/SRN and SRN/substrate interfaces to suppress high-order Mie resonant modes [48], [49]; (iii) compatible fabrication processes (*in-situ* deposition) for all materials.

## 2 The proposed multilayer all-dielectric metasurface

**Figure 1:**
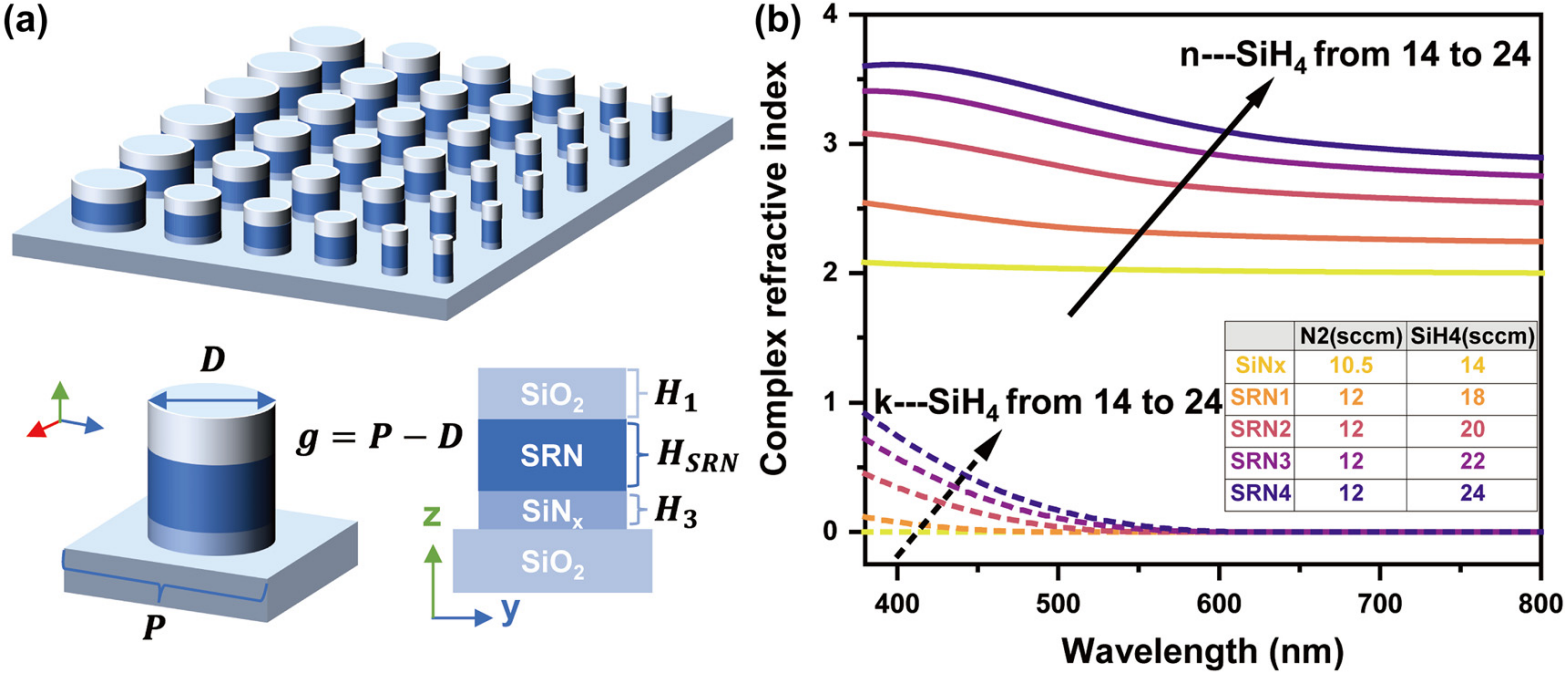
Multilayer all-dielectric metasurface design and optical properties. (a) Structural design of the multilayer all-dielectric metasurface under normally visible illumination. Each unit cell consists of a 100-nm thick (*H*_1_) SiO_2_ capping layer, a 150-nm thick (*H*_SRN_) SRN spacer layer, and a 50-nm thick (*H*_3_) SiN_*x*_ layer from top to bottom. (b) Dispersion characteristics of SiN_*x*_ and SRN in the visible spectral band (the ratios of SiH_4_ and N_2_ gases are measured in sccm).

The detailed schematic of the “color pixels” composed of the multilayer all-dielectric nanostructures on a glass substrate is shown in Figure 1(a). Each unit cell is made of a stacked 100-nm thick (*H*_1_) SiO_2_ capping layer, a 150-nm thick (*H*_SRN_) SRN spacer layer, and a 50-nm (*H*_3_) thick SiN_*x*_ layer from top to bottom with varying periods (*P*) of square lattices and diameters (*D*) of nanodisks. The diameter is varied from 100 nm to 300 nm to implement different metasurfaces working in the visible wavelength, while the gap size (*g*) presents the distance between adjacent nanodisks, and *g* = *P* − *D*. Figure 1(b) illustrates the optical properties in the visible region for the materials associated with SiN_*x*_, which are obtained by measuring films deposited on a glass substrate using the ellipsometer. SRN is attained with inductively coupled plasma chemical vapour deposition (ICP-CVD) at a low temperature of 300 °C, while the composition of SRN can be altered by adjusting the ratios of SiH_4_ and N_2_ gases, which are measured in standard cubic centimeters per minute (sccm). It is noted that SRN can provide a wide range of refractive indices, and it can be compatible with SiN_*x*_ and SiO_2_ in material fabrication processes.

**Figure 2:**
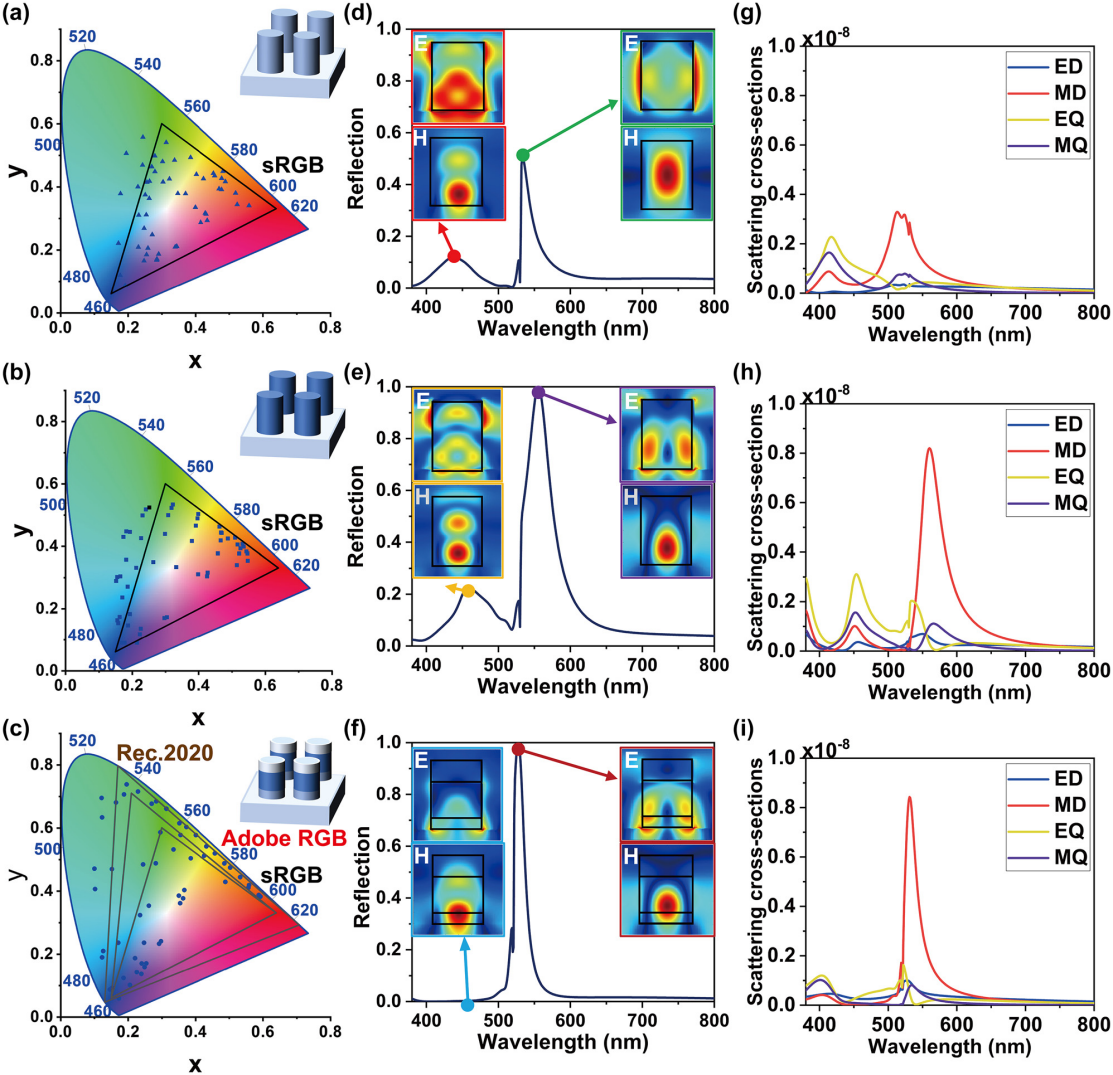
Color representations and optical characteristics for (a) all-SiN_*x*_ structures, (b) all-SRN structures and (c) multilayer all-dielectric structures (each shown 55 nanodisks with *P* from 300 to 400 nm in 10-nm step and *g* from 300 to 400 nm in 20-nm step). (d–f) Simulated reflection spectra of nanodisks with a period of 350 nm and a diameter of 200 nm. The insets show the electric field and magnetic field distribution of all-SiN_*x*_ structures at 437 nm (red box)/533 nm (green box), all-SRN structures at 458 nm (yellow box)/554 nm (purple box) and multilayer all-dielectric structures at 458 nm (blue box)/514 nm (brown box), respectively. (g–i) Multipolar decomposition of scattering cross-sections in terms of electric dipole (ED), magnetic dipole (MD), electric quadrupole (EQ), and magnetic quadrupole (MQ).

The multilayer all-dielectric design for the “color pixels” is critical to saturated colors. As the sketches show in Figure 2(a)–(c), 300-nm thick nanodisks with diameter *D* of 200 nm are arranged in a square lattice with period *P* of 350 nm (see Appendix A for more design details). All colors were calculated using MATLAB from spectral data and color-matching functions, as defined by CIE. The chromaticity coordinates (*x*, *y*) in the CIE 1931 color space were obtained using the following equations [3], [4]:

(1) $$\begin{array}{c} x=\frac{X}{X+Y+Z},\\ y=\frac{Y}{X+Y+Z}. \end{array}$$

*X*, *Y*, and *Z* are tristimulus values calculated using the following equations:

(2) $$\begin{array}{c} X=k\underset{\lambda }{\int }\bar{x}\left(\lambda \right)R\left(\lambda \right)S\left(\lambda \right)d\lambda ,\\ Y=k\underset{\lambda }{\int }\bar{y}\left(\lambda \right)R\left(\lambda \right)S\left(\lambda \right)d\lambda ,\\ Z=k\underset{\lambda }{\int }\bar{z}\left(\lambda \right)R\left(\lambda \right)S\left(\lambda \right)d\lambda . \end{array}$$

Here, $\bar{x}\left(\lambda \right)$ , $\bar{y}\left(\lambda \right)$ and $\bar{z}\left(\lambda \right)$ are the standard observer functions defined by the CIE. $R\left(\lambda \right)$ is the simulated transmittance or reflectance, *S*(*λ*) is spectral power distribution of the source light. Moreover, *Y* represents both the relative amount of green primary color and the luminance factor of the object and *k* is the normalizing constant and can be calculated from the light source as *Y* = 100:

(3) $$k=\frac{100}{\underset{\lambda }{\int }S\left(\lambda \right)\bar{y}\left(\lambda \right)d\lambda }$$

To assess the performance of the designed structures, simulations were conducted to obtain the CIE 1931 chromaticity coordinates for the structures shown in Figure 2(a)–(c), respectively.

Notably, the multilayer all-dielectric nanodisks exhibit significantly enhanced saturation enabling precise control of structural colors and enhancing performance. The corresponding CIE 1931 chromaticity coordinates visually demonstrate a more comprehensive coverage of hues and a drastic improvement in saturation. In order to evaluate the color gamut produced by the multilayer nanostructures, we calculated the color gamut area (0.1698) based on the color coordinates in the CIE diagrams, occupying 152 % of sRGB, 112 % Adobe RGB and 80 % Rec.2020 gamut (the area of the three standard gamut is shown in Table 2).Commercial finite element method software, COMSOL Multiphysics, is used for the numerical simulations, and a unit cell in the *x*–*y* plane is simulated, which perfectly matched layers are used in the *z*-direction. Figure 2(d)–(f) present the simulated reflection spectra of all-SiN_*x*_, all-SRN and multilayer all-dielectric nanostructures (a 100-nm thick SiO_2_ capping layer, a 150-nm thick SRN spacer layer, and a 50-nm thick SiN_*x*_ layer from top to bottom), respectively. Due to the relatively low refractive index of SiN_*x*_, the reflectivity of the all-SiN_*x*_ structure is low with a major peak at 533 nm and a minor peak at 437 nm. For all-SRN nanostructures, there is a minor peak at 458 nm, which seriously affects the monochromaticity of visible wavelength spectra. However, the reflection peak vanishes away in multilayer all-dielectric nanostructures. The insets of Figure 2(d)–(f) show the simulated electric and magnetic fields distribution at different resonant wavelengths. The high refractive index SRN material leads to localized magnetic field enhancement inside the dielectric nanostructures, enhancing the major peaks of reflection. As for the minor peaks, the evolution of electric and magnetic fields distribution around the metasurface indicates that the multilayer all-dielectric nanostructures can manipulate electric and magnetic resonant modes, suppressing the minor peaks of reflection.

**Table 2:** Plenty of color standard gamut in CIE 1931 diagram [4].

Color gamut	R	G	B	Area
sRGB	(0.640, 0.330)	(0.300, 0.600)	(0.150, 0.060)	0.1117
Adobe RGB	(0.640, 0.330)	(0.210, 0.710)	(0.150, 0.060)	0.1520
Rec. 2020	(0.708, 0.292)	(0.170, 0.797)	(0.131, 0.046)	0.2119

To further investigate the modulation role of multilayer all-dielectric designed, we decompose the multipolar modes of scattering cross-sections into electric dipole (ED), magnetic dipole (MD), electric quadrupole (EQ), and magnetic quadrupole (MQ) modes, as shown in Figure 2(g)–(i). The multipoles are generated by the induced polarization currents in the nanodisks. The current density $J\left(r\right)$ can be expressed as $J\left(r\right)=i\omega \varepsilon_{0}\left(\varepsilon_{r}-1\right)E\left(r\right)$ where $E\left(r\right)$ is the electric field distribution, *ɛ*_0_ is the permittivity of vacuum in free space, *ɛ*_*r*_ is the relative permittivity of the dielectric, and *ω* represents angular frequency. The Multipole moments is shown in Table 3.

**Table 3:** Multipole moments.

ED:	$P=\frac{1}{\text{i}\omega }\int d^{3}rJ$
MD:	$M=\frac{1}{2c}\int d^{3}r\left(r\times J\right)$
EQ:	$Q_{\alpha \beta }=\frac{1}{2\text{i}\omega }\int d^{3}r\left[r_{\alpha }J_{\beta }+r_{\beta }J_{\alpha }-\frac{2}{3}\left(r\cdot J\right)\right]$
MQ:	$M_{\alpha \beta }=\frac{1}{3c}\int d^{3}r\left[{\left(r\times J\right)}_{\alpha }r_{\beta }+{\left(r\times J\right)}_{\beta }r_{\alpha }\right]$

ED, electric dipole; MD, magnetic dipole; EQ, electric quadrupole; and MQ, magnetic quadrupole where *α*, *β* = *x*, *y*, *z* and *c* is light speed.

Using the multipole moments can obtain the sum of the contributions from different multipole moments [53], [54], [55]:

(4) $$\begin{array}{c} C_{\text{sca}}^{\text{total}}=C_{\text{sca}}^{ED}+C_{\text{sca}}^{MD}+C_{\text{sca}}^{EQ}+C_{\text{sca}}^{MQ}+\cdots \\ =\frac{k^{4}}{{6\pi \varepsilon }_{0}^{2}{\left|E_{\text{int}}\right|}^{2}}\left[\underset{\alpha }{\sum }{\left|P\right|}^{2}+{\left|\frac{M}{c}\right|}^{2}+\frac{1}{120}\underset{\alpha \beta }{\sum }\left({\left|kQ_{\alpha \beta }\right|}^{2}\right.\right.\\ \left.\left.\;\;\;+{\left|k\frac{M_{\alpha \beta }}{k}\right|}^{2}\right)+\cdots \;\right] \end{array}$$

where $C_{\text{sca}}^{\text{total}}$ represents the total scattering cross section. It’s the sum of the contributions from different multipole moments ( $C_{\text{sca}}^{ED},C_{\text{sca}}^{MD},C_{\text{sca}}^{EQ},C_{\text{sca}}^{MQ}\ldots$ ). $\left|E_{\text{inc}}\right|$ is the electric field amplitude of the incident plane wave, *k* is the wavenumber. For a periodic array of nanodisks, the contributions of multipoles to the scattering power can be expressed as [44], [45], [46], [47], [48], [49], [50], [51], [52], [53], [54], [56]:

(5) $$I_{\text{sca}}=\frac{2\omega^{4}}{3c^{3}}{\left|P\right|}^{2}+\frac{2\omega^{4}}{3c^{3}}{\left|M\right|}^{2}+\frac{\omega^{6}}{5c^{5}}Q_{\alpha \beta }Q_{\alpha \beta }+\frac{\omega^{6}}{20c^{5}}M_{\alpha \beta }M_{\alpha \beta }$$

In Figure 2(g) and (h), the calculations demonstrate the excitation of the EQ mode at a short wavelength, leading to multiple secondary peaks in all-SRN nanodisks. In contrast, the multilayer all-dielectric nanodisks effectively mitigate the influence of multipolar excitation, as depicted in Figure 2(i) (see Appendix B for details). Compared to dielectric materials with fixed refractive indices, SRN offers a wider range of refractive index options, thereby providing increased design flexibility for metasurfaces. Additionally, the *in-situ* deposition of SRN with SiN_*x*_ and SiO_2_ facilitates the creation of a superior heterogeneous interface.

In all-SRN nanostructures, the presence of certain resonant modes at short wavelengths leads to a reduction in the saturation of the generated structural colors. To address this issue, we utilized SiO_2_ and SiN_*x*_ as the capping and bottom layers, respectively, to achieve an index-matching condition and minimize the first peak of all-SRN nanostructures at 458 nm. While the refractive indices approximately satisfy the desired conditions of and, there may still be slight deviations from the ideal refractive index dispersion due to the sub-wavelength structure, resulting in deviations in the effective index due to Mie scattering effects.

The refractive index distribution of multilayer all-dielectric is shown in Table 4 (see Appendix A for more design details). As the refractive index distribution approaches a state of near-matching $n_{SiO_{2}}\approx \sqrt{n_{\text{SRN}}n_{\text{Air}}}$ with $H_{1}\approx \lambda /4\sqrt{n_{\text{SRN}}n_{\text{Air}}}$ and $n_{SiN_{x}}\approx \sqrt{n_{\text{SRN}}n_{\text{Sub}}}$ with $H_{3}\approx \lambda /4\sqrt{n_{\text{SRN}}n_{\text{Sub}}}$ , the sub-peak is effectively suppressed, resulting in a reflection spectrum characterized by a single peak and higher reflectance efficiency [57]. Such design strategy underlies the multilayer structure, enabling precise control of structural colors and enhancing performance (occupying 152 % of sRGB).

**Table 4:** The refractive index distribution (@ 500 nm).

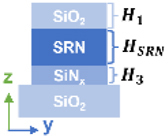	$n_{SiO_{2}}$	1.477	*H*_1_	100 nm
	$n_{SRN_{1}}$	2.357	*H*_SRN_	150 nm
	$n_{SiN_{x}}$	2.035	*H*_3_	50 nm

**Figure 3:**
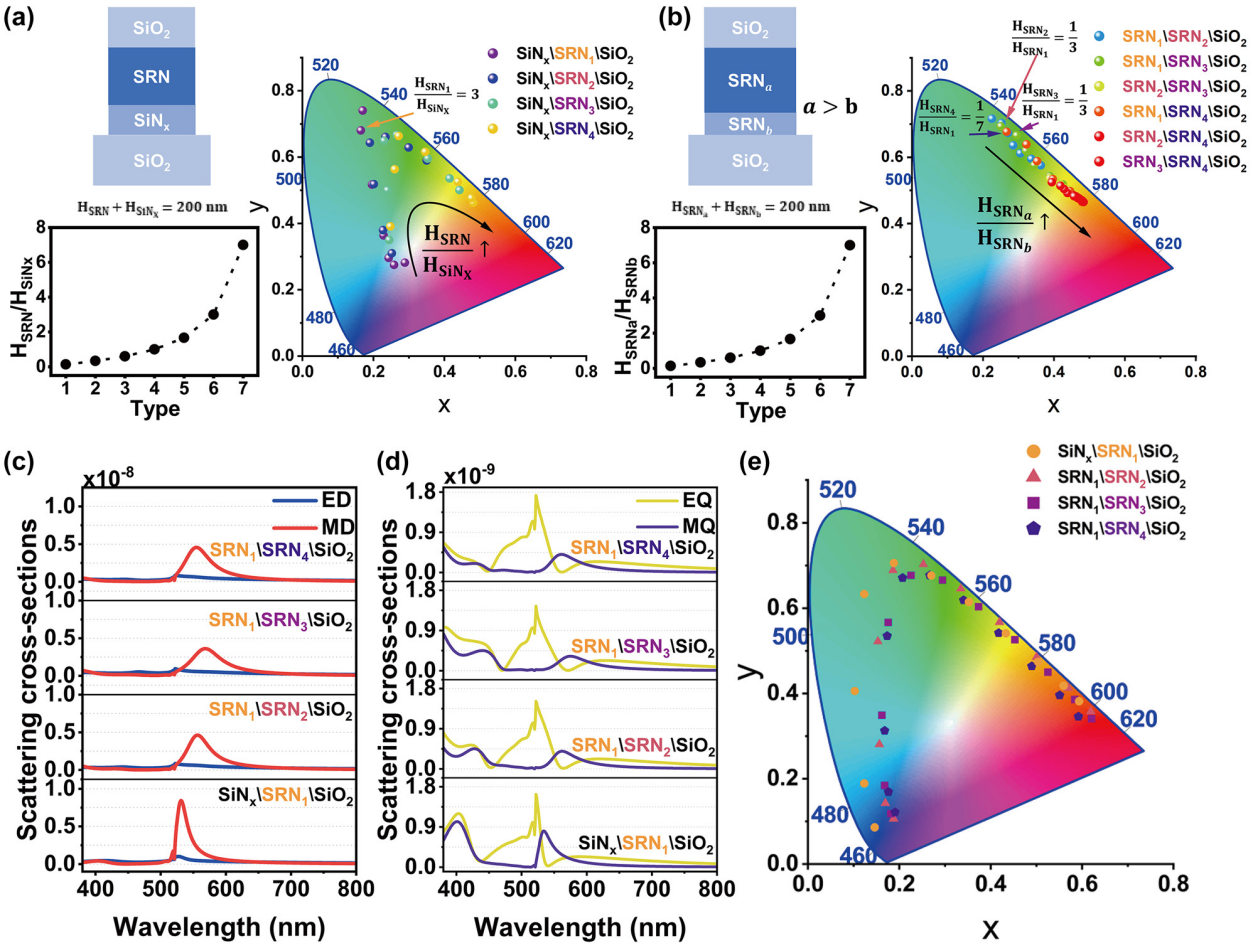
Influences on tunable refractive index SRN materials for the design of multilayer all-dielectric metasurface. (a) The 1931 CIE diagram for the structures of different refractive indices of middle layer (SRN_1_, SRN_2_, SRN_3_ and SRN_4_) with different SRN and SiN_*x*_ thickness ratios(in 25-nm step). (b) The 1931 CIE diagram for the structures of different refractive indices of middle and bottom layers (SRN_1_, SRN_2_, SRN_3_ and SRN_4_) with different SRN_*a*_ and SRN_*b*_ thickness ratios(in 25-nm step). (c, d) Calculated multipolar decomposition of scattering cross section distribution of ED, MD and EQ, MQ modes according to (a) and (b) (pointed by the arrow). (e) The 1931 CIE diagram for the structures optimized of different refractive indices of middle (SRN_1_, SRN_2_, SRN_3_ and SRN_4_) and bottom (SiN_*x*_ and SRN_1_) layers according to (a) and (b) (pointed by the arrow).

Figure 3 illustrates the stepwise optimization process of the refractive index distribution design for the multilayer all-dielectric metasurface based on tunable refractive index SRN materials, with a period of 350 nm, a diameter of 200 nm and the total thickness of the nanostructures set to 300 nm, the thickness of SiO_2_ layer set to 100 nm. In Figure 3(a), the middle layer consists of SRN with different refractive indices, while the bottom layer is SiN_*x*_. As the refractive index of SRN layer increases or the thickness ratio of the two layers (*H*_SRN_/*H*_SiN__*x*_) gradually increases, the color gradually shifts from blue to red.

To further investigate the influences on refractive index matching for structural color, the middle (SRN_*a*_ for higher refractive indices) and bottom layers (SRN_*b*_ for lower refractive indices) are defined in Figure 3(b), where subscripts a and b indicate the silicon content of SRN materials (*a* > *b*). The refractive index (*λ* = 500 nm) of SRN is shown in Table 5. The corresponding colors shift towards longer wavelength as the refractive index increases or the thickness ratio of the two layers (*H*_SRN*a*_/*H*_SRN*b*_) increases, always approaching the edge of the color gamut with good saturation, as demonstrated in Figure 3(b). Figure 3(c) and (d) show the ED, MD and EQ, MQ cross sections of the four refractive index combinations respectively (structural parameters indicated by arrows in Figure 3(a) and (b)). Among them, the color responses are dominated by MD modes, while high-order resonant modes (EQ and MQ modes) exhibit similar responses (spectral overlaps) in the short wavelength regions (see Appendix B for more design details). To demonstrate color rendering performance, we fixed gap size (*g*) at 150 nm with different scanned periods, and all four refractive index combinations broke through sRGB. The color gamut covered by SRN_1_, SRN_2_, SRN_3_, and SRN_4_ are 152 %, 142 %, 131 %, and 120 % of sRGB, respectively. Compared to SRN multilayer structures with lower refractive indices (SRN_1_), those with higher refractive indices (SRN_2_, SRN_3_ and SRN_4_) exhibit a decline in color performance, with a noticeable reduction in the covered color gamut. This degradation can be attributed to the rising silicon content in SRN materials, which not only increases the refractive index but also enhances absorption losses. These substantial absorption losses reduce reflectance (see Appendix B for more design details). Furthermore, with constant gap, the increased mutual coupling between adjacent elements results in significant broadening of the reflectance spectrum (see Appendix C for more design details).

**Table 5:** The complex refractive index of SRN (@ 500 nm).

	SRN_1_	SRN_2_	SRN_3_	SRN_4_
*n*	2.357	2.831	3.157	3.388
*k*	0	0.040	0.106	0.168

## 3 Experimental results

**Figure 4:**
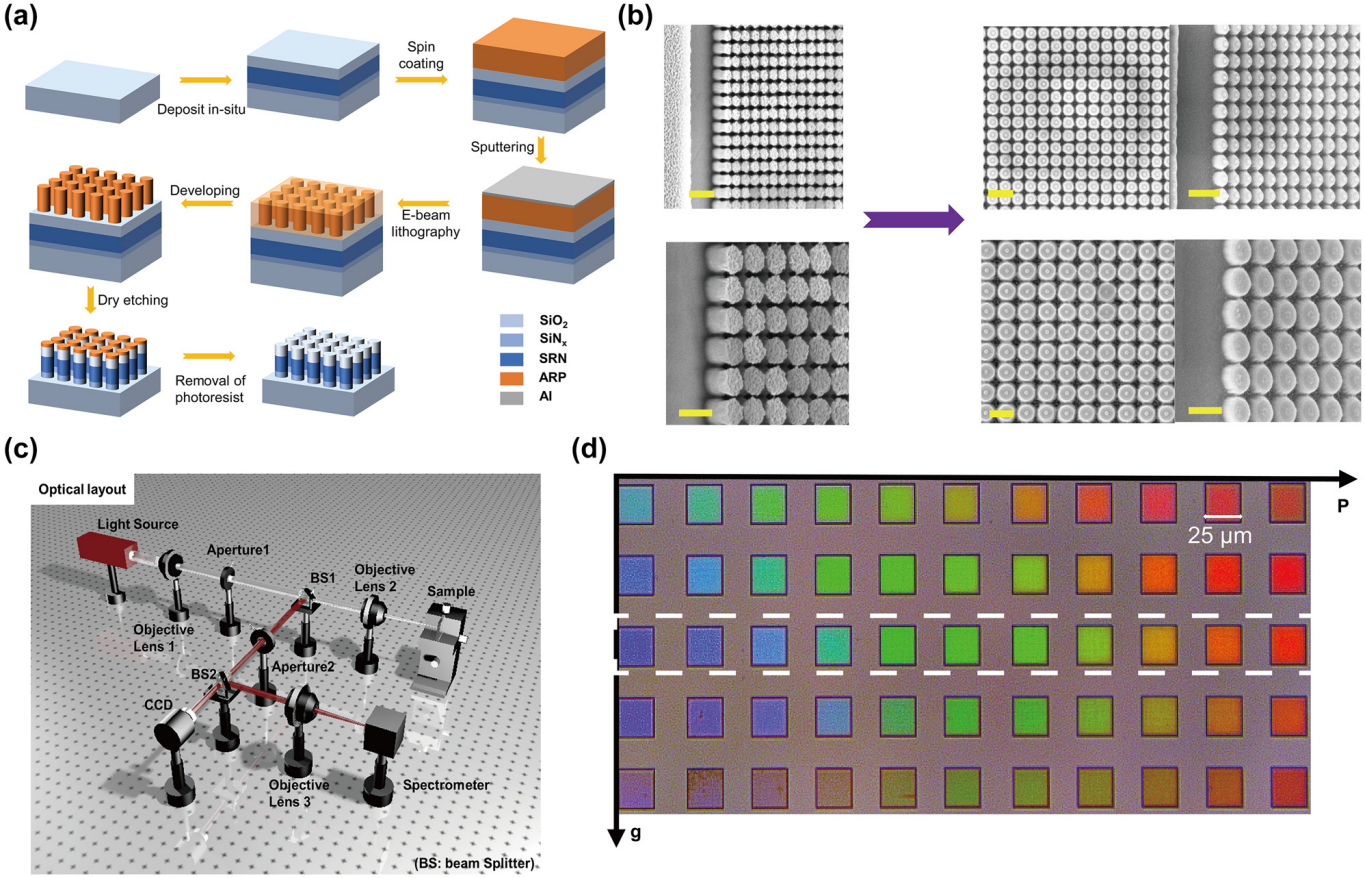
Fabrication and measurement of the multilayer SRN metasurface. (a) Schematic of the sample fabrication process. (b) The SEM images of the photoresist before etching and the multilayer SRN metasurface, scale bars are both 500 nm. (c) Schematic illustrating the optical setup for measuring spectra with spectrometer and capturing color images with CCD camera. (d) Measured color palette with varying periods and gaps. The size of each color palette is 25 μm × 25 μm.

The multilayer SRN metasurface is fabricated with ICP-CVD, electron-beam (E-beam) lithography and reactive ion etching (RIE). The fabrication procedure is schematically shown in Figure 4(a). First, SiN_*x*_, SRN_1_, and SiO_2_ were deposited on silica substrate successively in ICP-CVD (PlasmaPro System100, Oxford). A 50-nm thickness aluminum (Al) for overcoming the charge problem was deposited by ion sputtering (Q150T S Plus, Quorum) after a positive high-contrast e-beam resists AR-P 6200 (CSAR62) was coated on the materials layer. The layer was subsequently patterned through an E-beam lithography system (EBPG5000+, Riath). Then, the Al was removed by 5 % H_3_PO_4_ (at 50 °C). Using a gas mixture of CHF_3_ and SF_6_, multilayer nanodisks were etched in RIE (Plasmapro System 100, Oxford). The total size of the multilayer SRN metasurface is 25 μm × 25 μm for a color palette. The top-view and lateral-view scanning electron microscope (SEM) images in Figure 4(b) depict the photoresist before etching and the multilayer SRN metasurface, where the basic nanostructures are observed to be well preserved without any noticeable deformation throughout the fabrication process. The reflection measurement of the samples was conducted using an optical setup illustrated in Figure 4(c). We used a light source (SLS201L, Thorlabs) to provide the stable white light covers 360 nm–2,600 nm. The collimated linearly polarized light was directed onto the metasurface at a normal incident angle through a system of convex lens and beam splitter. Reflected by the metasurface, the incident light is split into two separate beams using a beam splitter. One beam is used to capture images by CCD camera, while the other beam is used to analyze the spectral properties of the modulated light by spectrometer. Figure 4(d) shows 55 regions in reflection mode, each with distinct periods and gap sizes. The color palette demonstrates a gradual transition in color, shifting from blue to red as the period *P* increases from 300 to 400 nm in 10 nm increments. In contrast, as the gap *g* size increases from 110 to 190 nm in 20 nm steps, there is a blue shift in the corresponding wavelength.

**Figure 5:**
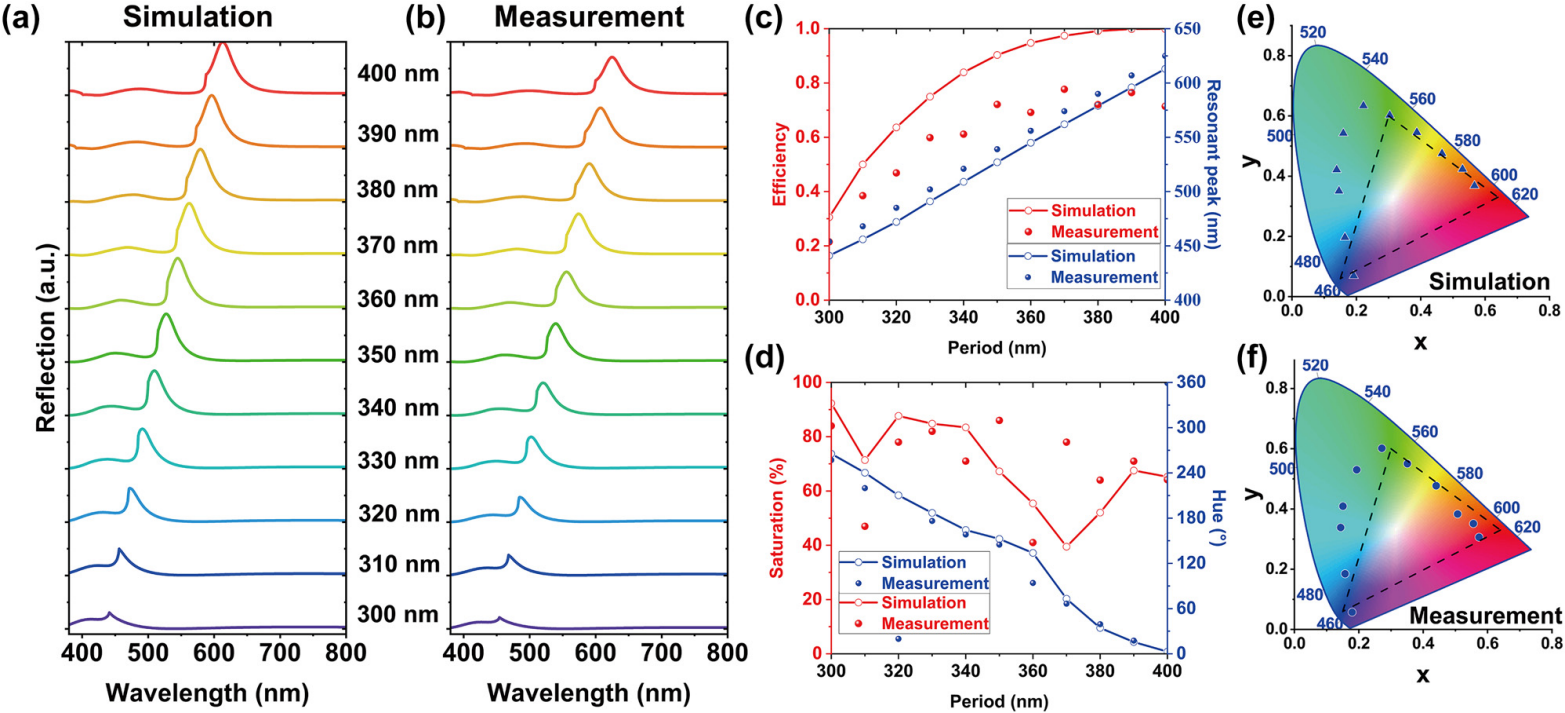
Simulated and measured results of pixels indicated by dashed boxes in Figure 3(d) with periods varying from 300 to 400 nm when the gap is fixed at 150 nm. (c, d) The comparison between simulated and measured results is shown for (c) the efficiency and resonant peak of the reflection spectra and (d) the hue and saturation of the reflected colors. The solid line corresponds to the simulated data, while the scattered dots represent the measured results. (e, f) The corresponding CIE 1931 chromaticity coordinates based on (c) simulated spectra and (d) measured spectra respectively.

To further investigate the performance of structural colors in experiments, we conducted a quantitative comparison between the simulated and measured spectra. Figure 5(a) illustrates the simulated spectra of multilayer all-dielectric nanostructures with varying periods from 300 to 400 nm while keeping the gap fixed at 150 nm. Due to the inherent differences between the designed thickness and the actual deposited thickness of the multilayer all-materials *in-situ* using ICP-CVD with only one recipe, we adjusted the simulated spectra based on thickness obtained from the optical film thickness gauge. Consequently, there are slight differences observed in the reflection spectra between the optimized results (a 100-nm thick SiO_2_ capping layer, a 150-nm thick SRN spacer layer, and a 50-nm thick SiN_*x*_ layer from top to bottom) in Figure 2(f) and the adjusted results (a 119-nm thick SiO_2_ capping layer, a 136-nm thick SRN spacer layer, and a 56-nm thick SiN_*x*_ layer from top to bottom) in Figure 5(a). As the period increases, the reflection peaks undergo a redshift from 441 to 613 nm in simulation and a redshift from 454 to 625 nm in measurement. Notably, all calculated reflection spectra exhibit no significant high peak in the short-wavelength region, which can be attributed to the modulation of multipolar modes between the refractive index-matched layers in the multilayer nanostructures. It is evident that the simulated spectra closely align with the corresponding measured spectra presented in Figure 5(b). For instance, when the period is fixed at 310 nm, the simulated resonance peak occurs at 456 nm with FWHM of 23 nm, while the measured peak is at 468 nm with FWHM of 25 nm, close to the FWHM of TiO_2_ metasurfaces [27]. Figure 5(c) provides a more intuitive visualization of the resonance peaks and efficiencies derived from the simulated and measured spectra. The slight shift in the resonance peak can be attributed to minor structural deviations caused by fabrication tolerances and the variation in the measurement. Furthermore, we compared the measured and simulated colors by evaluating the saturation and hue, as depicted in Figure 5(d). The measured colors exhibit high saturation and closely follow the trends observed in the simulated results. Additionally, Figure 5(e) and (f) demonstrate the color coordinates in the color gamut for both the simulated (occupying 152 % of sRGB) and measured (occupying 122 % of sRGB) results, respectively, clearly showcasing the overall red shift.

**Figure 6:**
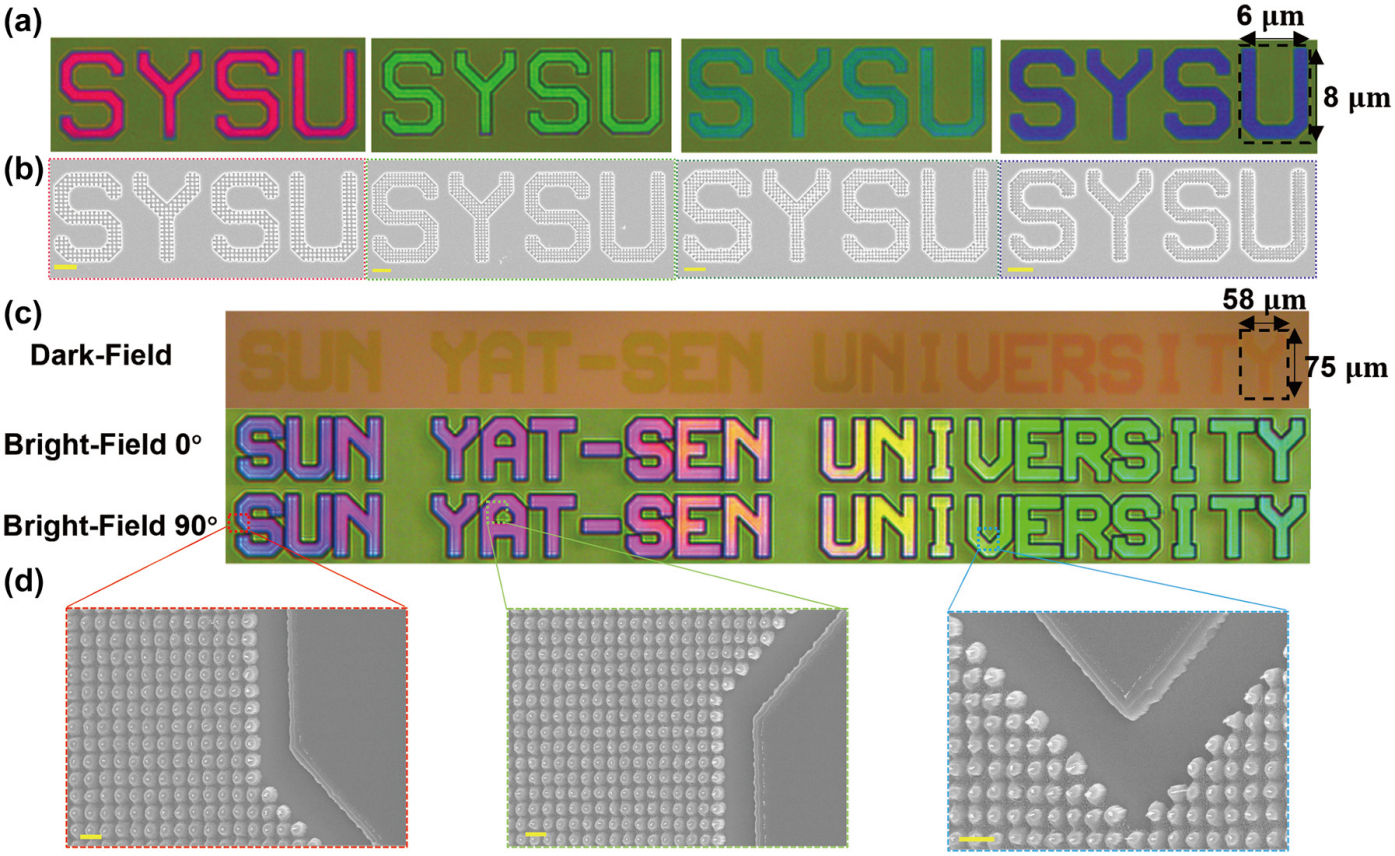
Colorful images printed by the SRN-based multilayer nanostructures. (a) The reflected images for fabricated “SYSU” patterns with four sizes. (b) Top-view SEM images for the patterns “SYSU” with period of 400 nm (highlighted by red box), 350 nm (highlighted by green box), 300 nm (highlighted by blue box) and 250 nm (highlighted by purple box) respectively. The scale bar is 2 μm. (c) The color-graded images for fabricated “SUN YAT-SEN UNIVERSITY” patterns of dark field, bright field with 0° and bright field with 90°, respectively. (d) Side-view (45°) SEM images for the patterns “S” with period of 400 nm and diameter of 290 nm (highlighted by red box), “A” with period of 380 nm and diameter of 210 nm (highlighted by green box) and “V” with period of 335 nm and diameter of 176 nm (highlighted by blue box), respectively. The scale bar is 1 μm.

The characters “SYSU” in Figure 6(a) were created using multilayer SRN metasurfaces with different nanodisk sizes and periods. These characters, with a size of 6 μm × 8 μm, are presented in four colors: red, green, blue, and purple. The top-view SEM images in Figure 6(b) provides valuable insights into achieving a high-resolution display with vibrant color pixels. The results suggest that for optimal performance, it is advisable to use multilayer SRN metasurfaces with a minimum size of approximately 1 μm × 1 μm (period of 250 nm). With pixels of this size, the resolution can reach 25,400 dpi, meeting the requirements of various image and display applications. Figure 6(c) showcases the structural color images of the metasurfaces in dark field, as well as *x*-polarized and *y*-polarized in bright field. The letters “SUN YAT-SEN UNIVERSITY” display gradient colors and are also constructed using nanodisks of varying sizes and periods. Each letter has a size of 58 μm × 75 μm. These metasurfaces exhibit vibrant and luminous color displays in both dark-field and bright-field settings. Furthermore, thanks to the symmetric unit design, the multilayer SRN metasurface is insensitive to polarization. Figure 6(c) provides SEM side-views of the letters “S” (highlighted by a red dashed box), “A” (highlighted by a green dashed box), and “V” (highlighted by a blue dashed box) as observed in Figure 6(b). Those findings emphasize the potential of SRN-based multilayer metasurfaces in delivering exceptional visual quality and intricate details.

## 4 Conclusions

In this study, we conducted a theoretical and experimental strategy on a multilayer all-dielectric metasurface based on SRN material, demonstrating its ability to generate vivid colors. The metasurface consists of three materials: SiO_2_, SRN, and SiN_*x*_, which can offer significant advantages in terms of fabrication feasibility and design scalability. By carefully designing the refractive index and thickness of each layer, we were able to achieve precise control over the optical response of the metasurface (∼25,400 dpi) and significantly improve color quality in the range of 400–500 nm, achieving a FWHM of 25 nm at 441 nm and an unprecedentedly large color gamut (about 152 % sRGB space in simulation and 122 % sRGB space in measurement). Overall, our study highlights the potential of multilayer all-dielectric metasurfaces for achieving vivid and high-performance color generation, while also emphasizing the practical advantages of the chosen materials in terms of fabrication compatibility.

## Figures and Tables

**Figure 7:**
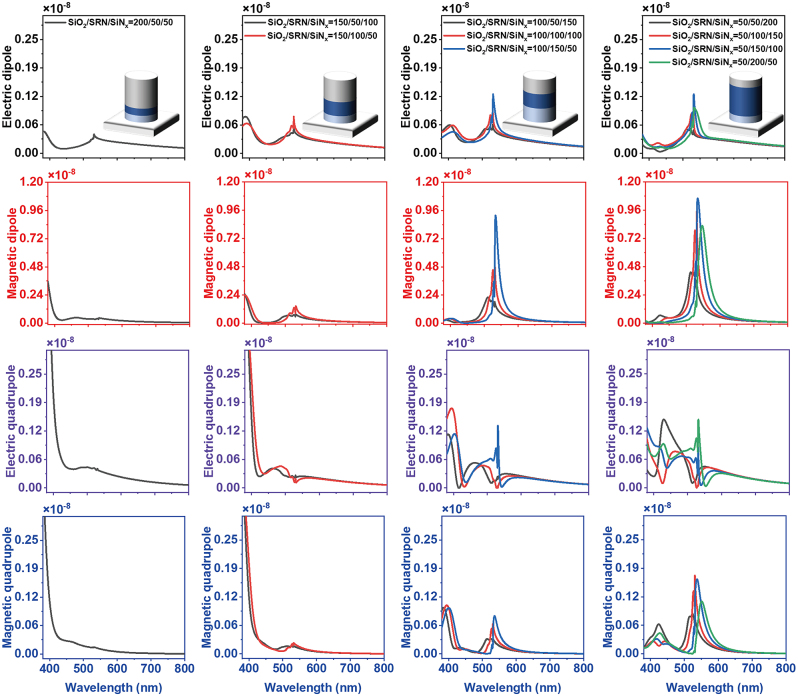
Multipolar decomposition of scattering cross-sections in terms of electric dipole (ED), magnetic dipole (MD), electric quadrupole (EQ), and magnetic quadrupole (MQ) in Table 6.

**Figure 8:**
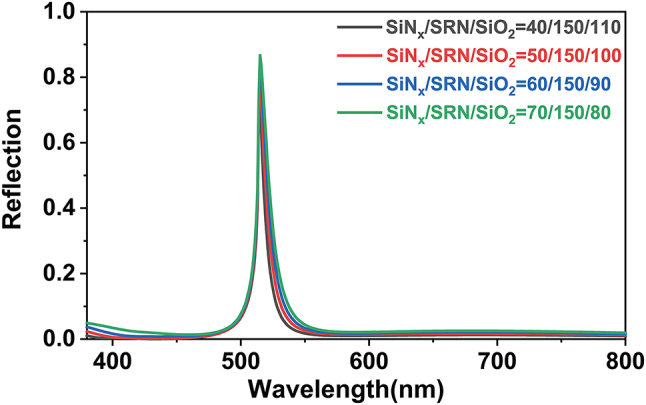
Simulated reflection spectra of nanodisks with a period of 350 nm and a diameter of 200 nm (the total thickness of three layers is set as 300 nm, SRN_1_ = 150 nm and SiN_*x*_ = 40/50/60/70 nm).

**Figure 9:**
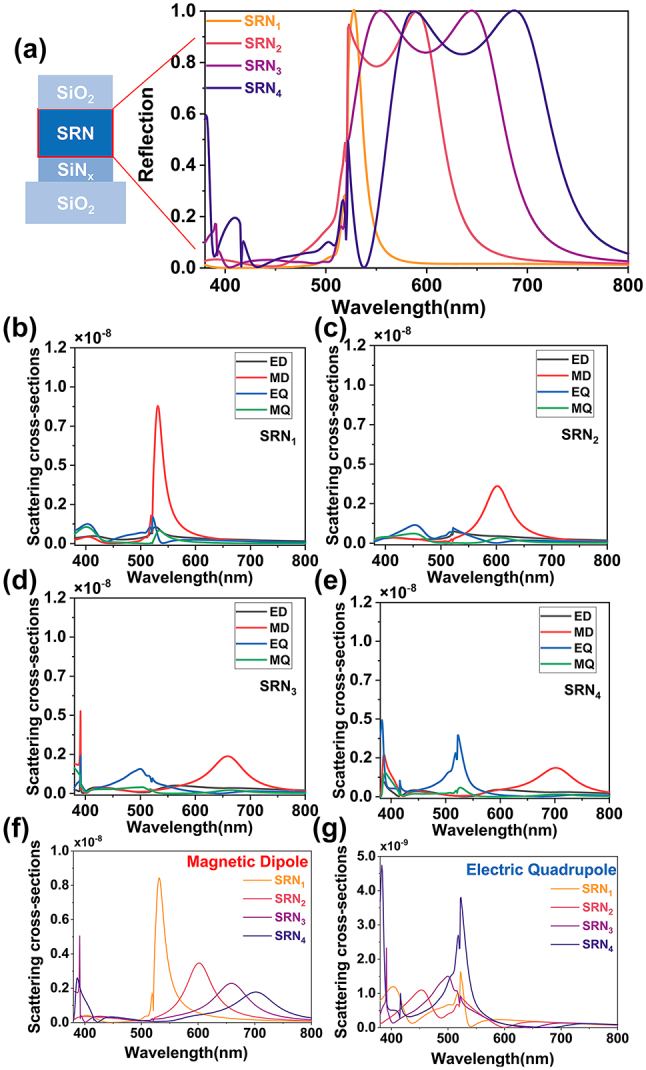
Optical properties of SRN-based metasurfaces and detailed analysis of multipolar scattering. (a) Simulated reflection spectra (b) absorption (calculated by *A* = 1 − *T* − *R*) and (c) The corresponding CIE 1931 chromaticity coordinates based on the with the middle layer (d) SRN_1_, (e) SRN_2_, (f) SRN_3_ and (g) SRN_4_ of nanodisks with a period of 350 nm and a diameter of 200 nm. Calculated multipolar decomposition of scattering cross section when the middle layer is (d) SRN_1_, (e) SRN_2_, (f) SRN_3_ and (g) SRN_4_ and (h) & (i) Magnifying distribution of MD and EQ mode for various material of middle layer.

**Figure 10:**
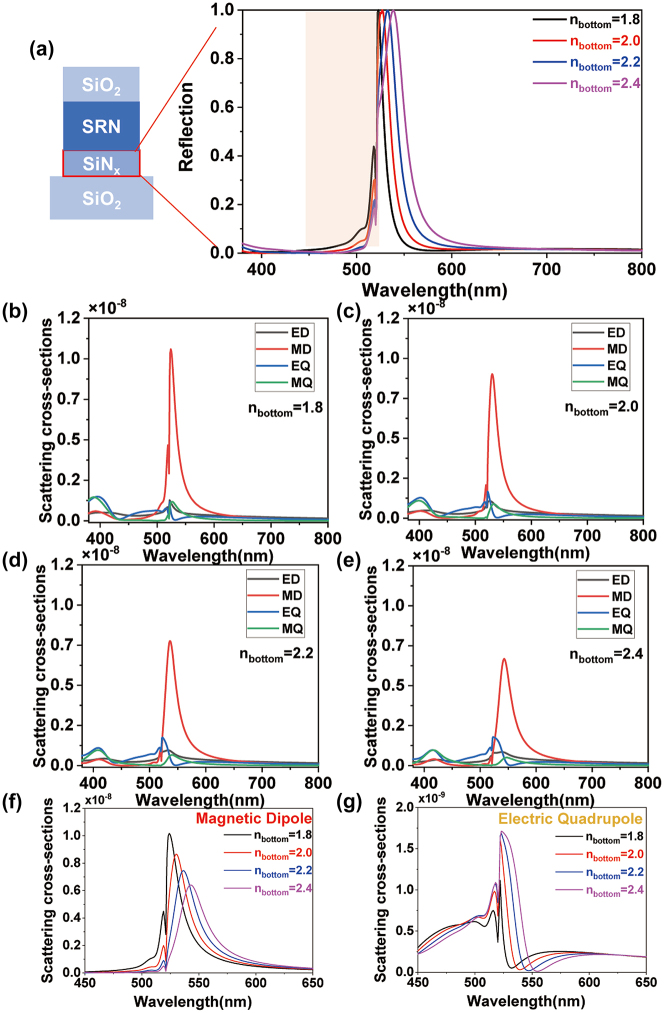
Optical properties of nanodisks with varied refractive indices. (a) Simulated reflection spectra with the refractive indices of bottom layer (b) 1.8, (c) 2.0, (d) 2.2 and (e) 2.4 of nanodisks with a period of 350 nm and a diameter of 200 nm. Calculated multipolar decomposition of scattering cross section when the refractive index of bottom layer is ((b) 1.8, (c) 2.0, (d) 2.2 and (e) 2.4). (g), (f) Magnifying distribution of MD and EQ mode for various refractive indices of bottom layer.

**Figure 11:**
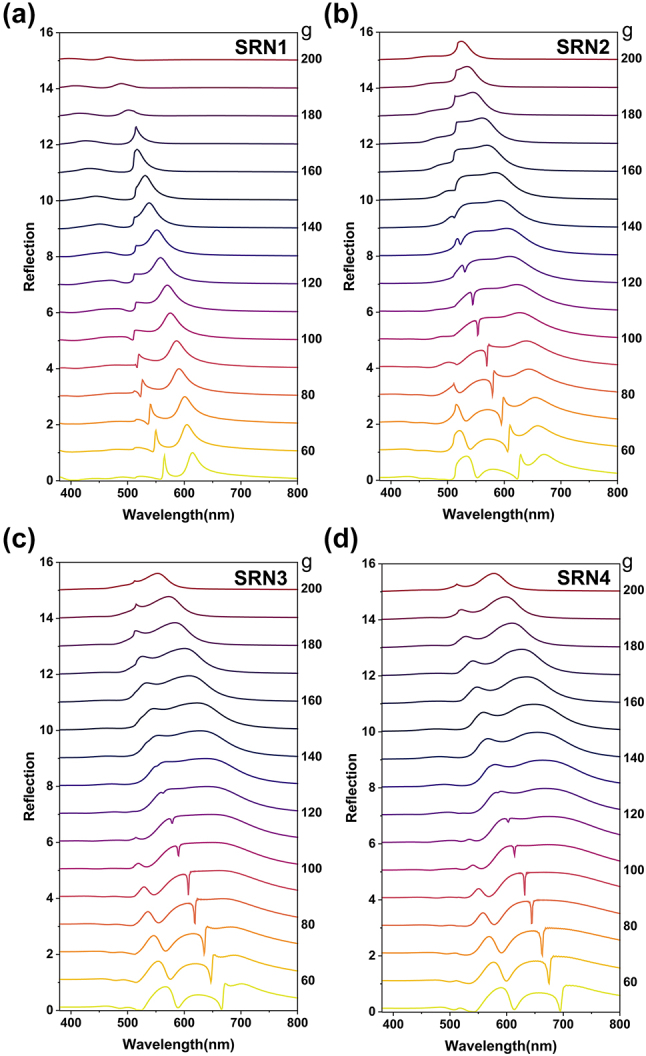
Simulated reflection spectra with (a) SRN_1_, (b) SRN_2_, (c) SRN_3_ and (d) SRN_4_ of nanodisks with a period of 350 nm and gap *g* from 50 nm to 200 nm (in 10-nm steps).

**Figure 12:**
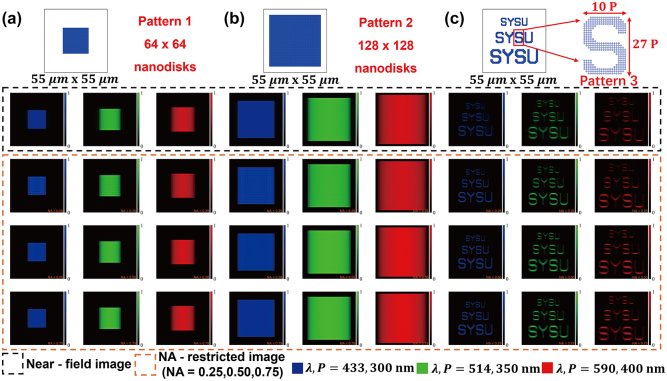
Imaging analysis of multilayer all-dielectric nano-patterns for near-filed and NA-restricted conditions. (a) The images of 64 × 64 pixels-based square patterns with different wavelengths and NA. (b) The images of 128 × 128 pixels-based square patterns with different wavelengths and NA. (c) The images of “SYSU” patterns with different wavelengths and NA. The pattern scale is related to resolution (the number of nanodisks) and the period of nanodisks (the top, middle and bottom layers were set as 100 nm SiO_2,_ 150 nm SRN_1_ and 50 nm SiN_*x*_, the gap *g* of 150 nm, respectively).

## Appendix A: Comparison among different thickness designs

We simulated different material layer thickness (in Table 6) to demonstrate our design strategy. First, the top and bottom layers are set as SiO_2_ (*H*_1_) and SiN_*x*_ (*H*_3_), the middle layer is set as SRN_1_ (*H*_SRN_) and the total thickness of three layers is set as 300 nm. From these 10 types different methods we conclude that the multilayer design is an efficient strategy to engineer multipole.

**Table 6:** Designed thickness of multilayer dielectric metasurfaces.

	SiO_2_ (*n* = 1.477)	SRN_1_ (*n* = 2.357)	SiN_*x*_ (*n* = 2.035)
Thickness (nm)	200	50	50
	150	50	100
	150	100	50
	100	50	150
	100	100	100
	100	150	50
	50	50	200
	50	100	150
	50	150	100
	50	200	50

As shown in Figure 7, the MD mode dominates the main reflectance peak, the insets illustrate the unit cell of multi-dielectric nanostructures. It is noteworthy that with changes in the structural parameters, the intensity of the MD mode does not always increase monotonically with the thickness increase of SRN material. When the *H*_SRN_ is set to 150 nm, the MD mode can reach its maximum in the parameter sets. Compared to the MD mode, other modes are relatively weaker. Interestingly, in the short wavelength region (particularly when the SiO_2_ thickness is set to 100 nm and 150 nm), EQ and MQ modes exhibit a peak corresponding to a dip. This phenomenon may suggest energy transfer between higher-order modes.

Note that the optimized wavelength *λ*_0_ is set as 500 nm, and $n_{SiO_{2}}\approx \sqrt{n_{\text{SRN}}n_{\text{Air}}}$ with $H_{1}\approx \lambda /4\sqrt{n_{\text{SRN}}n_{\text{Air}}}=81$ nm and $n_{SiN_{x}}\approx \sqrt{n_{\text{SRN}}n_{\text{Sub}}}$ with $H_{3}\approx \lambda /4\sqrt{n_{\text{SRN}}n_{\text{Sub}}}=66$ nm. Based on the previous analysis, the SRN thickness was set to 150 nm. We simulated several sets of structural parameters for SiN_*x*_ around 66 nm (as shown in Figure 8). The final material thickness distribution was determined as: a 100-nm thick SiO_2_ capping layer, a 150-nm thick SRN spacer layer, and a 50-nm thick SiN_*x*_ layer from top to bottom.

## Appendix B: Engineering for higher-order mode by varying index of middle layer and bottom layer

To investigate the effect of high-refractive-index components on the Mie scattering of multi-dielectric nanostructures, we simulated multi-dielectric nanostructures with different intermediate layer materials of 150 nm, as shown in Figure 9. The top and bottom layers were set as 100 nm SiO_2_ and 50 nm SiN_*x*_, respectively. The refractive indices of SRN_1_ – SRN_4_ gradually increase (from 2.36 to 3.39 @ 500 nm), and the absorption in the visible spectrum also increases (from 0 to 0.168 @ 500 nm) correspondingly. As the refractive index increases, the reflectance spectrum progressively broadens and shifts redwards (Figure 9(a)), and a corresponding red shift in absorption also occurs (Figure 9(b)), with an absorption peak consistently preceding each reflectance peak. Notably, within wavelengths shorter than 500 nm, absorption significantly increases with the silicon content in SRN and becomes non-negligible, which weakens the reflectance intensity of structural colors in the short-wavelength region. As the MD mode redshifts, its peak gradually decreases, whereas the EQ mode’s redshifted peak gradually increases, even surpassing the MD mode. With the same geometric parameters, SRN materials can achieve high reflectance. However, due to the different sensitivities of MD and EQ modes to changes in refractive index (MD being faster than EQ), the spectral broadening becomes increasingly severe.

To investigate the effect of the refractive index of the bottom layer on multipolar modulation, we simulated the multipolar decomposition of multi-dielectric nanostructures with different refractive indices of the SiN_*x*_ layer, as shown in Figure 10. The refractive index of the SiN_*x*_ layer varies from 1.8 to 2.4 (0.2 for a step). FWHM of the reflection spectra gradually increases with the increasing refractive index of the SiN_*x*_ layer (Figure 10(a)). Corresponding to the multipolar decomposition shown in Figure 10(b)–(e), the MD mode gradually redshifts and broadens. Additionally, as the refractive index of the SiN_*x*_ layer increases, the EQ mode also redshifts and intensifies, but at a slower rate than the MD mode. This slower redshift of the EQ mode is another reason for the increase in FWHM.

## Appendix C: Investigation of SRN composition on secondary peaks

In order to study the influence of adjusting SRN refractive index on reflection spectra, we simulated the reflection spectra of 300-nm SRN arrays with different component refractive indices under a single-material structure, with the period *P* fixed at 350 nm and the gap *g* varying from 50 nm to 200 nm, as shown in Figure 11. The refractive indices of SRN_1_-SRN_4_ gradually increase (from 2.36 to 3.39 @ 500 nm), and the absorption in the visible spectrum also increases (from 0 to 0.168 @ 500 nm) correspondingly. It has been noted that the spectral response of the proposed metasurface, which features periodically arranged SRN nanodisks, can be customized by adjusting the gap between neighboring elements. These gaps may facilitate mutual coupling to a certain extent, influencing the resonance characteristics of the metasurface. The proposed SRN metasurface exhibits higher efficiencies at longer wavelengths compared to shorter wavelengths. This is due to the enhanced resonances mediated by Mie scattering. In contrast, the reflection efficiency significantly decreases in the shorter wavelength region due to the combined effects of non-negligible optical extinction and less efficient scattering-mediated resonance. Under the same period, as the gap gradually decreases, SRN arrays with different refractive indices exhibit a gradual redshift and broadening of the reflection peak. When the gap is larger, the secondary reflection peaks gradually disappear, but the reflection intensity also decreases accordingly. It is worth noting that as the refractive index of SRN increases, the intensity of the primary reflectance peak also rises. This relationship holds until the refractive index reaches a certain threshold, beyond which the increase in intensity begins to plateau. Specifically, we observed that when the refractive index of SRN increased from 2.5 to 3.5, the primary reflectance peak’s intensity approached unity, indicating near-total reflectance at the resonant wavelength.

## Appendix D: Imaging analysis of multilayer all-dielectric nano-patterns

The near-field images are simulated with normal incident *x*-polarized Total-Field Scattered-Field (TFSF) source and PML conditions in Lumerical FDTD. The size of TFSF source is between simulation (55 × 55 µm^2^ with 20-nm mesh grid in *x*–*y* plane) and pattern regions to eliminate the effects of background reflection and PML boundary diffraction. It is noted that the diffraction effects can be observed from the edges of patterns (Figure 12, especially “SYSU” patterns).

To further evaluate the diffraction effects for patterns with different scales, the near fields can be decomposed into a series of plane waves to reconstruct NA-restricted images (the plane waves with angles outside of the NA are discarded). For the cases of NA = 0.25, the patterns become clearer at short wavelengths with larger scales (higher resolution). For the cases of larger NA, the serious diffraction effects can’t be observed from large-scale patterns at the same wavelengths. Thanks to sub-wavelength designed nanodisks (higher-order diffraction suppression), the diffraction effect caused by nano-pattern can be weakened through high-resolution pattern and large-NA imaging system. Therefore, the multilayer all-dielectric metasurface can support large-scale and high-resolution displays.
